# The Effect of Group Reflection on Nursing Students' Spiritual Well-being and Attitude Toward Spiritual Care: a randomized controlled trial

**DOI:** 10.17533/udea.iee.v37n1e09

**Published:** 2019-01-06

**Authors:** Marzieh Momennasab, Zahra Shadfard, Azita Jaberi, Seyed Saeed Najafi, Fakhrozaman Naeemi Hosseini

**Affiliations:** 1 B.Sc, M.Sc, Ph.D. Associate professor, Nursing department, Shiraz University of Medical Sciences, Shiraz, Iran. email: momennasab@sums.ac.ir Shiraz University of Medical Sciences Iran momennasab@sums.ac.ir; 2 B.Sc, M.Sc. student. Student research committee, Shiraz University of Medical Sciences, Shiraz, Iran. email: shadfard_or@yahoo.com Shiraz University of Medical Sciences Iran shadfard_or@yahoo.com; 3 B.Sc, Ph.D. School of Nursing and Midwifery, Community Based Psychiatric Care Research Center, Shiraz University of Medical Sciences, Shiraz, Iran. email: a_jaberi@sums.ac.ir Shiraz University of Medical Sciences Iran a_jaberi@sums.ac.ir; 4 B.Sc, M.Sc. Instructor. Nursing department, School of Nursing and Midwifery, Shiraz University of Medical Sciences, Shiraz, Iran email: najafisa@sums.ac.ir. Corresponding author. Shiraz University of Medical Sciences Iran najafisa@sums.ac.ir; 5 Assistant professor. PhD of distance education, Center of Excellence for Electronic Learning in Medical Sciences, Shiraz University of Medical Sciences, Shiraz, Iran. email: fnaeemi@sums.ac.ir Shiraz University of Medical Sciences Iran fnaeemi@sums.ac.ir

**Keywords:** students, nursing, spirituality, attitude, control groups, nursing, estudiantes de enfermería, espiritualidad, actitud, grupos control, atención de enfermería., estudantes de enfermagem, espiritualidade, atitude, grupos controle, cuidados de enfermagem

## Abstract

**Objective.:**

To investigate how group reflection about spiritual care affects nursing students' spiritual well-being and attitude toward spirituality and spiritual care.

**Methods.:**

This was a randomized controlled trial conducted on 63 second-year nursing students who were studying at Nursing and Midwifery Colleges in Shiraz and Jahrom, both located in south of Iran. The students were randomly divided into an intervention (*n*=30) and a control (*n*=33) group. The study data were collected using the Spiritual Well-Being Scale and Spirituality and Spiritual Care Rating Scale before and after the intervention. The intervention consisted in four sessions of group reflection based on the scenarios related to spiritual care. The control group was given a related lecture in one session.

**Results.:**

A significant difference was found between the two groups' means in spiritual well-being scores after the intervention compared to before that. Likewise, a significant difference was observed in the intervention group students' total scores of attitude before and after the intervention.

**Conclusion.:**

Group reflection improved the nursing students' spiritual well-being and their attitude toward spirituality and spiritual care compared with control group.

## Resumen

**Objetivo.:**

Investigar cómo la reflexión grupal sobre el cuidado espiritual afecta el bienestar espiritual y la actitud de los estudiantes de enfermería hacia la espiritualidad y el cuidado espiritual.

**Métodos.:**

Este fue un ensayo controlado aleatorio realizado en 63 estudiantes de segundo año de dos escuelas de enfermería y partería en Irán. Los estudiantes se dividieron aleatoriamente en los grupos de intervención (*n*=30) y de control (*n*=33). Los datos del estudio se recopilaron utilizando *la Escala de Bienestar Espiritual* y la *Escala de Calificación de Espiritualidad y Cuidado Espiritual* antes y después de la intervención. La intervención consistió en cuatro sesiones de reflexión grupal basadas en escenarios relacionados con el cuidado espiritual. El grupo de control recibió solamente una conferencia relacionada con el tema.

**Resultados.:**

Se encontró una diferencia significativa entre las medias de los dos grupos en cuanto a las puntuaciones de bienestar espiritual después de la intervención en comparación con la de la preintervención. Asimismo, se observó una diferencia significativa en las puntuaciones totales de actitud de los estudiantes del grupo de intervención antes y después de la intervención.

**Conclusión.:**

La reflexión grupal mejoró el bienestar espiritual de los estudiantes de enfermería y su actitud hacia la espiritualidad y el cuidado espiritual en comparación con el grupo de control.

## Resumo

**Objetivo.:**

Investigar como a reflexão grupal sobre o cuidado espiritual afeta o bem-estar espiritual e a atitude dos estudantes de enfermagem para a espiritualidade e o cuidado espiritual.

**Métodos.:**

Este foi um ensaio controlado aleatório realizado em 63 estudantes de segundo ano de duas escolas de enfermagem e de parteiras no Irã. Os estudantes se dividiram aleatoriamente nos grupos de intervenção (*n*=30) e de controle (*n*=33). Os dados do estudo se recopilaram utilizando *a Escala de Bem-estar Espiritual* e a *Escala de Qualificação de Espiritualidade e Cuidado Espiritual* antes e depois da intervenção. A intervenção consistiu em quatro sessões de reflexão grupal baseadas em cenários relacionados com o cuidado espiritual. O grupo de controle recebeu somente uma conferência relacionada com o assunto.

**Resultados.:**

Se encontrou uma diferença significativa entre as médias dos dois grupos em quanto às pontuações de bem-estar espiritual depois da intervenção em comparação com a da pré-intervenção. Assim mesmo, se observou uma diferença significativa nas pontuações totais de atitude dos estudantes do grupo de intervenção antes e depois da intervenção.

**Conclusão.:**

A reflexão grupal melhorou o bem-estar espiritual dos estudantes de enfermagem e sua atitude à espiritualidade e o cuidado espiritual em comparação com o grupo de controle.

## Introduction

Health is a general concept that includes physical, social, cultural, emotional, and spiritual dimensions. So a holistic view must be considered in providing health care.(1) Since spirituality is an essential component of health and well-being and one aspect of holistic care, professional nursing governing bodies stressed on providing spiritual care in nursing practice.(2) Spiritual care is both essential and unique and answers basic questions related to the pain, suffering, and death and supporting patients with finding meaning, purpose and hope.(3) Although spiritual care is an essential, accepted, and required dimension of nursing practice(4) and nurses have intention and motivation for providing spiritual care,(5) research findings demonstrated many of them are not able to provide it adequately(2) and this integral aspect of holistic care often ignored.(6)

Therefore, it is necessary to teach spirituality as a major component of holistic care.(7) Since students begin to learn the basic concepts and principles of holistic care during their education, they should be taught the spiritual aspects of care and the ways to internalize spiritual values during their course of education by means of proper methods. Although many researchers have claimed that increased knowledge of spirituality and spiritual well-being enhances nurses' capacity to provide spiritual care, not many studies have addressed the strategies to improve nurses' spiritual well-being and attitude toward spiritual care. A review of the literature also revealed that little has been written about teaching nurses regarding spiritual care.(8) The methods used to teach spirituality often include exploratory techniques, such as brain storming, questioning, case-studies analysis, small group discussions, critical thinking about personal spirituality, and presenting students with possible scenarios.(8) Reflection is a modern teaching approach that has been proved to be effective in increasing nurses' knowledge and skills in clinical situations, and can be used to teach the spiritual aspects of care(9). Group reflection is a kind of teamwork where different perspectives can be used to learn about and clarify an issue. It also helps individuals improve their reflection skills under the influence of others.(10)

Spiritual care education in Iranian nursing is subtle, ambiguous, infor mal, and nonprogrammable. A recent study in Iran reported that due to the lack of relevant contents in the nursing curriculum, educators are try ing to be role models for their students; in turn, nursing student also experience and understand spiritual care informally with continuous presence in clinical practices.(11) It can be said that nurses' knowledge of spiritual care in Iran is poor and they need specific informa tion on how to meet patients’ spiritual needs. Reflective groups have a huge potential to help nurses and nursing students learn about spiritual care and critically consider their everyday practice regardless of the practice setting, specialty, level of experience.(12) Reflective group working as a means of developing an individual’s abilities, skills and knowledge mirrors other shifts in thinking.(13) Nursing students learn that in order to gain from a practice experience, they will benefit from critically analyzing the situation and applying their newly gained perspective to future experiences.(12) Considering poor spiritual care in Iranian nursing students and teaching benefits of reflexive group, the present study aims to investigate the impact of teaching spiritual care through group refection on nursing students' spiritual well-being and their attitude towards spirituality and spiritual care. 

## Methods

### Design, Setting and Participants.

**Figure 1:**
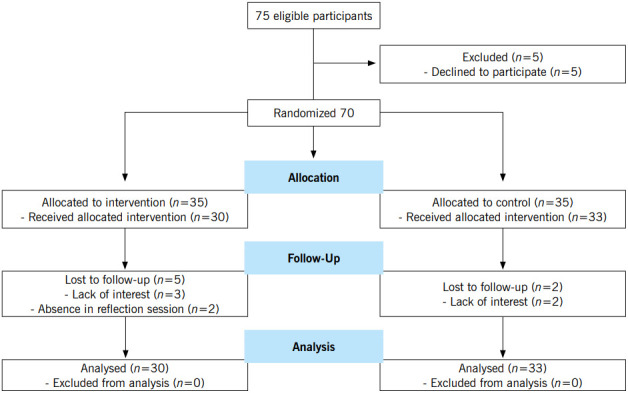
Flow Diagram of the study

This study was a randomized controlled trial conducted at Nursing and Midwifery School in Shiraz and Nursing School in Jahrom, both located in south of Iran. In spring semester of 2014, all of nursing students who were in third and fourth semester in two mentioned schools were included in the study. Out of the 75 second-year nursing students, 70 (36 students from Shiraz and 34 students from Jahrom) were willing to participate in the study. Exclusion criteria were missing more than one reflection sessions and unwillingness to continuing participation in the study. In each school participants were randomly divided into two groups by block randomization with a random sequence of 2 or 4 block sizes. During the study, 7 students (5 from the interventional group and 2 from the control group) were excluded according to exclusion criteria. Eventually, the collected data from 63 students (30 in the intervention group and 33 in the control group) were analyzed (Figure 1). 

### Data Collection.

The study data were collected using a demographic information form and two standard questionnaires. The first one was Spiritual Well-Being Scale (SWBS), designed by Ellison (1982), which is the most popular and widely used scale to assess the individuals’ subjective perception of their quality of life in relation with spirituality, as perceived through religious and existential dimensions. It consisted of 20 items which could be responded through a 6-point Likert scale, ranging from "I totally agree" to "I totally disagree". Thus, the total score of the questionnaire ranged from 0 to 120. This scale included two subscales, namely religious well-being and existential well-being. Each part, in turn, included 10 items whose scores ranging from 0 to 60.(14) The validity and reliability of the questionnaire were verified in the previous studies. Its overall reliability is approved with Cronbach’s alpha 0.82. In the present study, the Persian version of the questionnaire was used which has been reported to have appropriate content validity and internal consistency (α=0.7)(15) The second instrument was Spirituality & Spiritual Care Rating Scale (SSCRS) which used to evaluate the participants' attitude. SSCRS was originally developed by McSherry et al. (2002) for investigating nurses’ beliefs and values in relation to the nine fundamental areas including hope, meaning and purpose, forgiveness, beliefs and values, spiritual care, relationships belief in a God, or deity, morality, creativity and self expression.(16) This 17-item, Likert scale instrument was designed to “discover and explore nurses' understanding of and attitudes toward the concepts of spirituality and spiritual care”. It is a valid and reliable measure of spirituality/spiritual care with the intended sample. It has been used in over 42 studies in 11 countries demonstrating consistent levels of reliability & validity with Cronbach's alpha scores ranging from 0.64 to 0.84.(17) There are 17 statements scored on a 5-point scale from ‘strongly agree’ to ‘strongly disagree’. A high overall score indicates a broader view of spirituality (i.e. inclusive of both religious and existential elements) and spiritual care (i.e. facilitating religious rites/rituals as well as addressing patients' need for meaning, value, purpose, peace and creativity). The content validity and reliability of the Persian version of the scale which was used in this study was verified previously. , In the day of data collection, the study objectives and procedures were explained to the students. Subsequently, the students who were willing to participate in the study were asked to sign the written informed consent. Afterwards, the students were asked to complete SWBS and SSCRS. After all, the participants were randomly divided into an intervention and a control group.

### Intervention.

The students in the intervention group were divided into 3 groups in Shiraz and 2 groups in Jahrom nursing school. Each group attended 4 two-hour sessions of reflection. Two scenarios related to spiritual care were presented in each session for the groups to reflect over. The subjects of these scenarios were search for meaning and purpose, relationship with God, others, and environment, forgiveness, prayer and religious rituals, hope instillation, and family and nurse presence. These scenarios were developed based on academic resources such as nursing textbooks and the common events in clinical wards according to researchers’ experiences. Then their content was revised and confirmed by five nursing professors. Group reflections were done according to the Gibbs’ reflective cycle which is often used as a user-friendly framework for reflection and derives from Kolb’s principle of experiential learning.(12) This cycle consists of 6 stages, namely description of the event, expression of feelings, evaluation, analysis, conclusion, and action plan. In the reflection sessions that are directed by the researcher, after reading each scenario according to stages of Gibbs’ model following questions were asked: What happened? What are your feelings? What’s good and bad about the event? What sense do you make of this event? What have you learnt from this scenario? What would you do differently? The students in the control group, on the other hand, attended a two-hour session during which the concepts of spirituality and spiritual care were presented through common educational method -a formal lecture- by the researcher. There is not any group discussion in this session, but the students asked their questions. Two weeks after the last reflection and lecturing sessions, the participants in both groups completed SWBS and SSCRS. Finally, the entire students were given access to the scenarios and the contents of the lecture presented to the intervention and control groups.

### Ethical considerations.

This study was approved by the Ethics Committee of Shiraz University of Medical Sciences (No. 93-6987). The participants after receiving oral explanation about the study objectives and procedures signed consent. The students were also assured that refusal to participate in or withdrawal from the study would not affect their academic evaluation. Also anonymity and confidentiality of information were guaranteed.

### Data analysis.

The collected data were analyzed using the SPSS statistical software. Descriptive statistics were used to describe the students' demographic characteristics. Besides, paired t-test was employed to compare each group’s mean scores before and after the intervention. Independent t-test was also used to compare the mean differences of the two groups’ scores. In addition, Pearson's correlation test was applied to determine the relationship between the students' spiritual well-being and spiritual attitude.

## Results

### Demographic characteristics

Among the participants, 42 were female (66.7%). The mean age of the participants was 20.84±1.7 years and their age ranged from 19 to 25 years. The mean ages of the students in the intervention and control groups were 20.56±1.74 and 21.1±1.21 years, respectively, and the students’ age and sex distribution and marital status was almost identical in the two groups (*p*>0.05). 78% of the students were single. In addition, the students' Grade Point Averages (GPA) in the intervention and control groups were 16.95±0.69 and 16.92±0.70, respectively, but the difference was not statistically significant.

### Spiritual well-being

At the beginning of the study, no significant difference was observed between the two groups regarding the students' total scores of spiritual well-being as well as its sub-scales; i.e., religious well-being and existential well-being (Table 1). At this stage, the students' total score of spiritual well-being was 95.69±12.29, which was a high score. However, at the post-test, a significant difference was found between the intervention and control groups concerning the total score of spiritual well-being. Besides, the results of independent t-test showed that the mean differences of the two groups' well-being mean scores in pre- and post-tests were statistically significant. The difference was also significant in the case of the existential well-being subscale. Although, this was not the case regarding the religious well-being subscale (Table 1). 

**Table 1:** Comparison of the intervention and control groups regarding the mean scores of spiritual well-being and its subscales

Comparison	Baseline Mean (SD)	End of intervention Mean (SD)	Difference Mean (SD)	Paired t-test p-value
Total spiritual well-being				
Intervention group	95.83 (13.36)	102.36 (11.33)	6.5 (10.92)	0.0003
Control group	95.57(11.43)	95.12 (13.86)	0.45 (12)	0.829
*Between group p-value*	*0.935*	*0.028*	6.5 (10.92)	
Religious well-being subscale				
Intervention group	51.16 (7.11)	52.56 (6.13)	1.4 (5.23)	0.154
Control group	51.39 (6.50)	50.54 (8.07)	0.84	0.519
*Between group p-value*	*0.894*	*0.271*		
Existential well-being subscale				
Intervention group	44.6 (8.94)	49.86 (7.17)	5.2 (8.65)	0.0003
Control group	43.63 (8.09)	44.69 (9.07)	1.06 (8.52)	0.479
*Between group p-value*	*0.625*	*0.014*		

### Attitude toward spirituality and spiritual care

The results revealed no significant difference between the two study groups with respect to the students' total scores of attitude toward spirituality and spiritual care and their mean scores of the subscales in the pre-test (Table 2). At this stage, the students’ mean score of attitude was 57.31±9.74 which signified their semi-satisfactory attitude. In the intervention group, the total mean score of the scale and the mean score of attitude toward spirituality subscale differed significantly between the pre-test and the post-test. However, no significant difference was observed regarding attitude toward spiritual care subscale (Table 2). The study findings demonstrated no significant correlation between the nursing students' spiritual well-being and attitude toward spirituality and spiritual care and their age and sex. Also, Pearson's correlation test showed no statistically significant relationship between the students' spiritual well-being and their attitude.

**Table 2:** Comparison of the nursing students' attitudes toward spirituality & spiritual care and its dimensions in the two groups before and after the intervention

Spiritual well-being	Baseline Mean (SD)	End of intervention Mean (SD)	Difference Mean (SD)	Paired t-test p-value
Total Spirituality & spiritual care				
Intervention group	56.83 (11.52)	60.76 (7.04)	4.20 (10.42)	0.047
Control group	57.75 (4.97)	60.51 (9.75)	2.84 (8.45)	0.068
*Between group p-value*	*0.715*	*0.939*		
Spirituality subscale				
Intervention group	28.30 (5.98)	30.76 (4.36)	2.46 (5.87)	0.029
Control group	29.15 (4.57)	30.90 (6.27)	1.75 (5.85)	0.094
*Between group p-value*	*0.532*	*0.918*		
Spiritual care subscale				
Intervention group	28.53 (6.62)	30.00 (4.89)	1.46 (6.47)	0.220
Control group	28.60 (4.93)	29.60 (4.82)	1 (5.04)	0.263
*Between group p-value*	*0.961*	*0.749*		

## Discussion

The results of the present study showed that after group reflection on spiritual care, the mean score of the intervention group was higher than that before the intervention and differed significantly from that of the control group. These results confirmed contribution of group reflection to the students' spiritual well-being. According to a previous study, by providing spiritual care, nurses could enhance their own spiritual well-being, as well.(18) The students' existential well-being, as a sub-category of spiritual well-being, was most significantly affected in the current study. Existential well-being is based on one's relationship with others, environment, and oneself(19) and forms an important part of spiritual care. Establishing an effective relationship with patients is the key to spiritual care;(20) hence, this concept was considered in most of the scenarios in this study. This may account for the fact that the students' existential well-being was more affected by reflection compared to the other dimension of spiritual well-being. On the other hand, individuals’ religion and religious well-being are influenced by such factors as culture and personal beliefs and family background(21) and, thus, are not easily affected by short-time interventions. Yet, the current study findings showed the nursing students' high levels of spiritual well-being. Other studies have also indicated that Iranian students' spiritual well-being is relatively high.(17) This advantage can be used to improve nursing students' spiritual care skills in Iran.

The results of the present study demonstrated that group reflection improved the students' overall attitude towards spirituality and spiritual care. Through group reflection, the students were exposed to clinical situations which they had not considered before and by reflecting on and discussing the situations in groups, the learners' feelings and attitudes were affected. Evidence has approved that group reflection sessions influenced students' perceptions and conceptions more than other educational approaches. In fact, reflective teaching methodology allows ongoing mentoring of students and enables transferring learning into clinical practice related to spiritual care.(22) According to Lindberg's study, group reflection in the field of profession and professional skills increased nurses' professional satisfaction.(23) In our study, the students developed a better attitude towards spirituality. In other words, the technique of group reflection positively affected the students' spiritual attitude. This finding is similar to that of the study by Baldacchino where teaching spirituality and spiritual care had positive effects on the learners' personal dimensions and internal spirituality.(8) Although the students' scores of attitude to spiritual care were higher after the intervention, the difference was not statistically significant. In other words, group reflection did not significantly influence the students' attitude towards spiritual care. This might be attributed to the traditional methods students encounter in clinical wards every day. In addition, students' attitude towards spiritual care can be affected by barriers to spiritual care, such as inadequacy of nurses, emphasizing the routines, and poor communication skill.(24)

In the current study, the control group students' mean scores of overall attitude to spirituality and spiritual care and its two subscales increased in the post-test after attending a lecturing session; however, the difference was not statistically significant. According to another study, lecture can be effective in teaching spirituality and spiritual care.(8) Thus, when using student-centered approaches is not possible, traditional approaches are suggested to be employed. It is interesting to note that in the present study, most of the students' mean scores of attitude toward spirituality and spiritual care were semi-satisfactory, while another study in Iran indicated the satisfactory attitude of most of the nurses toward spirituality and spiritual care.(17) This difference can be explained by the differences between nursing students' and practicing nurses' experiences. The students in this study were in the second year of education and had little clinical experience. It has been proved that more experienced nurses have better attitudes towards spirituality and spiritual care.(25) It is also believed that nursing students' clinical experience is not enough for them to obtain a professional attitude which includes the attitude toward spirituality and spiritual care.(26) Moreover, the topic of spirituality is not treated separately in the nursing curriculum,(8) while these students need to be taught the spiritual aspects of care which are essential to their preparation for their future professional roles and provision of holistic care. It is also important to employ proper educational strategies to teach this aspect of clinical care.(26) Furthermore, teaching spirituality to large groups of learners might not be effective and, consequently, it has been suggested that spirituality be taught to small groups through workshops. Additionally, active student-centered strategies have been proved to be more useful.(27) Other studies also showed that different teaching methods and strategies have be used based on students' learning preferences.(28) Reflection not only enhances learners’ cognitive skills, but also reduces the gap between theory and practice.(6) Therefore, it has been recommended as an effective method in teaching spirituality.

According to the previous studies, there is a close relationship between nurses' internal spirituality and tendency to provide spiritual care. In other words, the higher the nurses' spirituality, the more they will try to provide spiritual care.(29) However, the results of Pearson's correlation test in this study showed no significant relationship between the nursing students' spiritual well-being and their attitude towards provision of spiritual care (*p*=0.74). It should be noted that students' attitude toward spirituality and spiritual care is affected not only by their spiritual well-being, but also by such factors as culture, religious beliefs, and personal traits.(25) One of the limitations of this study was the limited number of participants, which makes the results difficult to generalize. Therefore, future studies are suggested to be performed on larger groups of students in various years of study and degrees, practicing nurses, and students of other majors. Another limitation of the study was using non-native questionnaires. Since spirituality is influenced by cultural and social factors, employing the questionnaires designed in other cultures can cause a limitation. Of course, the questionnaires used in this study were in Persian and their validity and reliability had been verified.

The study results showed that teaching spiritual care through group reflection enhanced the nursing students' spiritual well-being and improved their attitude towards spirituality and spiritual care. Accordingly, this active student-centered approach, which can easily be performed, is recommended to be used in nursing students’ course of education. Teaching spiritual care through group reflection will increase students' sensitivity to patients' spiritual needs in their future professional practice. Also, by improving their spiritual well-being, students can positively influence the provision of holistic care both during their studies and after graduation.
